# Tapentadol vs oxycodone/naloxone in the management of pain after total hip arthroplasty in the fast track setting: an observational study

**DOI:** 10.1186/s40634-019-0204-6

**Published:** 2019-07-29

**Authors:** Tiziana D’Amato, Federica Martorelli, Giorgia Fenocchio, Vincenzo Simili, Elizaveta Kon, Berardo Di Matteo, Marco Scardino

**Affiliations:** 1 Department of Anesthesia, Humanitas Research Hospital, Rozzano, Milan, Italy; 2 Department of Biomedical Sciences, Humanitas University, Via Manzoni 113, 20089 Rozzano Milan, Italy; 3 Humanitas Clinical and Research Center, Via Manzoni 56, 20089 Rozzano Milan, Italy; 4 First Moscow State Medical University - Sechenov University, Moscow, Russia; 5 Center for functional and biologic reconstruction of the Knee Humanitas Clinical and Research Institute, Via Manzoni 113, 20089 Rozzano, Italy

**Keywords:** Fast track, Multimodal analgesia, Tapentadol, Naloxone/oxycodone, Total hip replacement, Pain management

## Abstract

**Background:**

In recent years, joint replacement surgery has gradually progressed towards the fast-track model, and early rehabilitation immediately after surgery is regarded fundamental for optimal recovery of function: the aim of the present study is to describe the efficacy in perioperative management of pain in patients undergoing total hip replacement surgery and treated with tapentadol or oxycodone/naloxone in combination with ketoprofene.

**Methods:**

Single-center retrospective study on patients with moderate-severe pain, referred to total hip replacement. Patients received either tapentadol (100 mg/twice-daily post-surgery – treatment group) or oxycodone/naloxone (10 mg/5 mg post-surgery – control group) plus ketoprofen 100 mg/ twice daily. Supplemental analgesia (paracetamol 1 g or morphine 0,1 mg/kg sc) was provided if needed. Pain at rest and pain during movement were evaluated on a daily basis for 4 days post-op, after which patients were usually discharged. All adverse events were reported and compared between the two groups.

**Results:**

106 patients were analyzed in the tapentadol group and compared to 105 patients treated with oxycodone/naloxone. Both pain intensity at rest and upon movement were significantly lower in the tapentadol group at all follow-up times (*p* < 0.001). Throughout T1-T4, supplemental analgesia was needed by significantly less tapentadol patients compared to the control group. Similarly, regarding side effects, a significantly higher occurrence of post-op nausea, vomit, itching and constipation was observed in the control group (*p* < 0.001 in all cases).

**Conclusion:**

Results from the present study support the use of tapentadol in combination with ketoprofen for the management of moderate-severe pain in the setting of major orthopedic surgery, given its effectiveness in reducing pain intensity, and its satisfactory tolerance.

## Background

In recent years replacement surgery has gradually progressed towards the Scandinavian fast-track model (Rodriguez-Merchan, 2015), which aims to facilitate a quick rehabilitation by ensuring that the patient reaches surgery in clinically and psychologically optimal conditions. Eliciting such a model involves tackling two major issues: (i) the management of moderate-severe chronic pain (generally classified as a “mixed” pain given the coexistence of both nociceptive and neuropathic components) reported by most patients waiting for orthopedic intervention, and (ii) the need to counteract the onset of collateral effects typical of new opioid-based analgesics as well as other negative stimuli which could affect post-operative rehabilitation. Since these issues apply to most types of orthopedic surgery, they also represent a paramount problem in the field of total hip replacement (THR), where early rehabilitation immediately after surgery is fundamental for the optimal recovery of functionality and its associated conditions (such as the recovery of autonomous ambulation and psycho-social wellbeing).

In such a setting, tapentadol appeared to be an appropriate solution for these yet unsolved issued, and its use has therefore been adopted within our surgical department for all patients undergoing THR with chronic moderate-severe pain.

From a pharmacological point of view tapentadol PR (prolonged release – commercial name: Palexia®, Grunenthal Srl, Italy) is the first of a new class of drugs, namely MOR-NRI: a novel analgesic with a dual mechanism of action, featuring a μ opioid receptor agonist (MOR) and a noradrenaline reuptake inhibitor (NRI). This single molecule thus combines the analgesic actions of both mechanisms in a synergistic fashion, while eliciting a minimal serotonergic effect (Tzschentke et al., 2014; Langford, 2016; Coluzzi & Ruggeri, 2014). Taken separately, the analgesic effects of each mechanism are quite modest, yet together they are able to produce a greater effect comparable to that of a more traditional, single mechanism opioid such as morphine or oxycodone (Schroder et al., 2011). Conversely such a synergistic effect does not elicit more profound or more frequent collateral effects (nausea, vomit, constipation) (Langford, 2016; Cowan et al., 2014; Afilalo et al., 2010). Moreover, differently from any other product, it does not require CYP enzyme activation and does not interfere with hematic clotting functions. These features therefore avoid surgery-associated risks and the need to suspend pain management treatment (as is seen with the use of FANS) (Tzschentke et al., 2014; Langford, 2016; Pergolizzi et al., 2016; Sanchez de Aguila et al., 2015). Additionally, since it does not depend on enzymatic activation, use of the molecule reduces the effect of patient variability in pain management, as is observed with the use of other drugs (Langford, 2016; Schroder et al., 2011; Cowan et al., 2014).

In the literature several works document the effectiveness of Tapentadol in chronic musculo-skeletal pain (Coluzzi & Ruggeri, 2014; Sanchez de Aguila et al., 2015; Hartrick et al., 2009). A multicenter study by Gigliotti et al. (Gigliotti et al., 2014) evaluated 97 patients affected by severe joint pain (either caused by musculo-skeletal symptoms, neuropathic syndromes or rheumatic disease). The patients were treated with tapentadol PR 50–250 mg/ twice daily and were evaluated over a time period of > 2 months, with results showing a mean pain-relief score of 7.1 (from 0 to 10). In a prospective study by Notaro et al., 27 patients with severe chronic back pain, who were not responsive to other analgesic treatments, were treated with tapentadol PR 100–500 mg and observed for six months. The patients showed significant improvement in the short term, with a 27% reduction in NRS by T3, and a 44% reduction in NRS at rest after 9 days (Notaro, 2017). A randomized study by Afilalo et al. compared the efficacy and the safety of tapentadol to placebo and oxycodone in patients with moderate to severe chronic pain due to knee osteoarthritis. Results showed that tapentadol’s effect on pain was similar to that of oxycodone, but with fewer collateral effects (Afilalo et al., 2010).

The purpose of the present observational study was to evaluate the efficacy of tapentadol in combination with ketoprofen in controlling post-operative pain in patients undergoing fast track THR surgery by comparing it to another commonly used analgesic drug, i.e. oxycodone/naloxone.

## Methods

### Patients selection

The present study was a single-center observational retrospective study involving two groups of patients undergoing hip replacement surgery (THA) between 2016 and 2018: one treated according to our previous standard of care for fast track surgery, i.e. oxycodone/naloxone + ketoprofen (control group), and the other treated with tapentadol + ketoprofen (treatment group). Inclusion criteria were: age between 60 and 80; ASA score between I-III; presence of hip arthritis requiring total hip replacement (THR) surgery. Exclusion criteria were: patients with infections, rheumatoid arthritis, fractures, and severe neuropathic diseases, or patients undergoing chronic cortisone treatment for any other medical reason.

Medical records of all patients treated with THR between 2016 and 2018 were therefore analyzed, and 106 patients in the tapentadol group and 105 in the naloxon group were ultimately included in the present analysis.

### Treatment

The patients from the treatment group underwent a pre-operatory check-up with an anesthesiologist approximately 30 days before scheduling the THR procedure. All patients received combined spinal-epidural anaesthesia with chirocaine 0,25% 7 mg via intrathecal injection and by epidural chirocaine 0,75% 40 mg, morphine 2 mg, and atropine 0,13 mg, prior to surgery. Following surgery, 100 mg of tapentadol was administered twice daily (Sanchez de Aguila et al., 2015) for 4 days (after which patients were normally discharged), whereas in the control group therapy consisted in administering oxycodone/naloxone 10 mg/5 mg twice daily per os; both treatments were in combination with ketoprofen 100 mg twice daily. The rescue dose for both groups consisted of paracetamol 1 g when NRS < 3 and a morphine 0,1 mg/kg SC if NRS > 3. For what concerns post-op nausea and vomit (PONV) management, a standard prophylaxis was performed by intra-venous administration of 4 mg Ondasentron + 10 mg Metochlopramide + 125 mg metilprednisolone + Ranitidin 50 mg diluted in 500 ml of saline solution or ringer acetate, before anesthesia induction. Further doses of 4 mg ondansetron i.v. were administered in the post-op period upon patient’s request in case of need.

### Clinical outcome evaluation

The study’s primary objective was the assessment of pain, both at rest and during movement, evaluated by NRS every day after surgery for up to 4 days (T1 = day 1, T2 = day 2, T3 = day 3, T4 = day 4), after which patients were generally discharged from the hospital. The study’s secondary objective involved assessing the daily occurrence of collateral effects (nausea, vomit, vertigo, drowsiness, constipation).

### Statistical analysis

The descriptive variables were reported as mean, standard deviation, median, minimum and maximum, whereas discrete and nominal variables were reported in a contingency table with rates being written as percentages. Results were reported as percentage and CI, with α = 0,05 one-tail. The 11-score NRS was analyzed via analysis of variance on repeated measures with multiple comparisons between measurements. The threshold value for statistical significance was *p* < 0,05 (5%). Between-group differences for qualitative and categorical variables were evaluated via Pearson’s t-test or Fisher’s test with Yeats when appropriate. Whereas differences for quantitative variables were evaluated via Student’s T-test or Mann-Whitney-Wilcoxon test, based on data distribution. All statistical tests were performed with Stata 14 (4905 Lakeway Drive College Station, Texas 77845 USA). Statistical significance for all analyses was considered *p* < 0.05.

#### Ethical considerations

The study was submitted to the Institutional Review Board and carried out in accordance with the principles outlined in the Helsinki Declaration. Data was retrospectively collected, double checked by two authors, and anonymized to create a dataset for statistical analysis: all patients included in the present study gave consent to the use of their data for scientific purposes.

## Results

The two study groups involved 105 patients within the control group and 106 patients within the tapentadol group, with both featuring similar characteristics in terms of gender distribution, age, BMI, and comorbidities. The most prevalent age group (32%) was represented by females between the ages of 70 and 80, which is in fact representative of the typical population admitted within our Orthopaedic Department for the specific procedure under consideration.

**Fig. 1:**
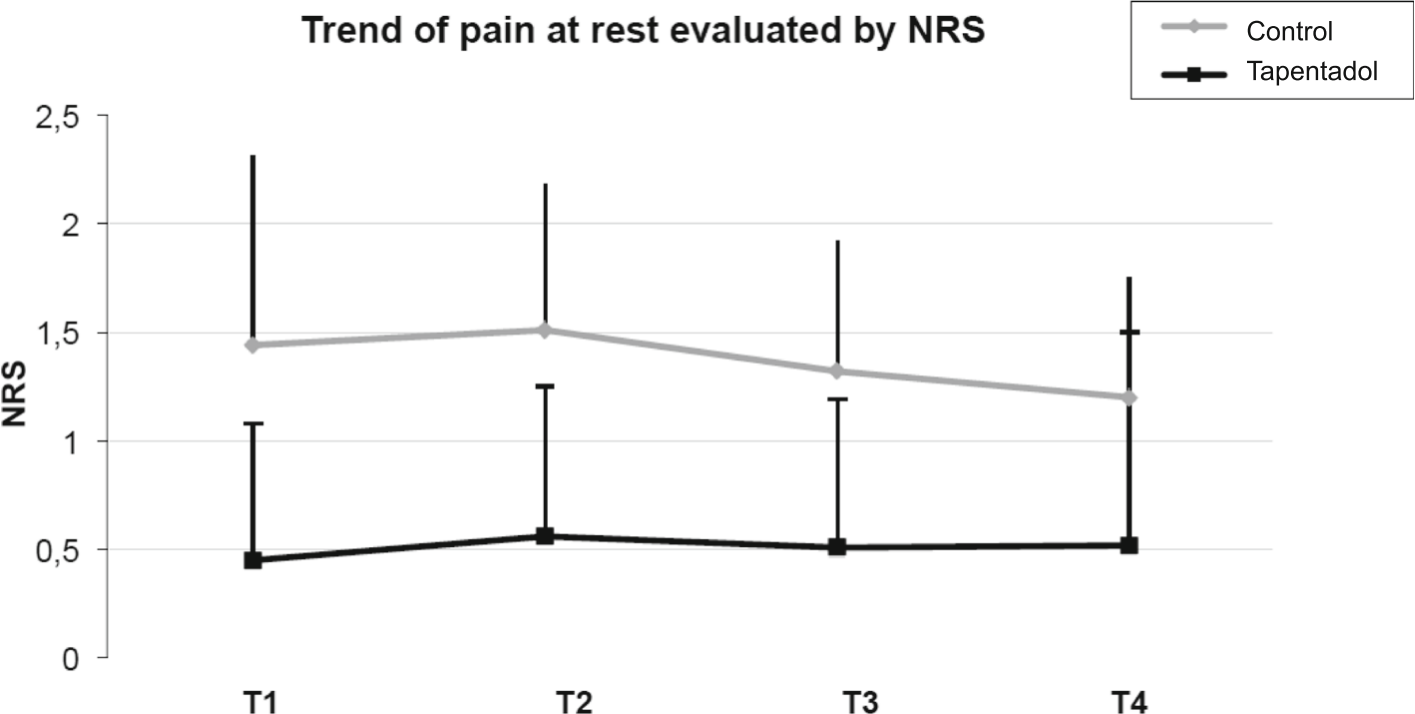
Trend of pain at rest evaluated by NRS in both treatment groups (Grey: Control group; Black: Tapentadol)

Primary endpoint Inter-group differences were all statistically significant (*p* < 0.001). As for changes to the NRS, pain was statistically higher in the control group throughout all four days (*p* < 0.001). In the tapentadol group specifically, NRS at rest went from 0.5 on T1 to 0.6 on T2, and then decreased and remained constant at 0.5 until T4. Conversely NRS in the control group started at 1.4 on T1, and slightly increased to 1.5 on T2, and then decreased to 1.3 on T3, and 1.2 on T4 (Fig. 1 and Table 1).

**Table 1:** Values of rNRS and mNRS in the two treatment groups at the various intervals

Variables	Control *n* = 105	Tapendatol *n* = 106	*p* value
rNRS T1	1.44 (± 0.87)	0.45 (± 0.23)	*p* < 0.001
rNRS T2	1.51 (± 0.67)	0.56 (± 0.29)	p < 0.001
rNRS T3	1.32 (± 0.60)	0.51 (± 0.28)	p < 0.001
rNRS T4	1.20 (± 0.55)	0.52 (± 0.38)	p < 0.001
mNRS T1	2.97 (± 1.32)	1.61 (± 1.42)	p < 0.001
mNRS T2	2.92 (± 1.36)	1.92 (± 1.32)	p < 0.001
mNRS T3	2.71 (± 1.24)	1.40 (± 1.17)	p < 0.001
mNRS T4	2.37 (± 1.17)	1.63 (± 1.14)	p < 0.001
Rescue Analgesic, n (%)			
T1	38 (36%)	8 (8%)	p < 0.001
T2	33 (31%)	16 (15%)	p < 0.001
T3	26 (25%)	12 (11%)	p < 0.001
T4	22 (21%)	10 (9%)	p < 0.05

rNRS: numeric rating score at rest; mNRS: numeric rating score while moving

T1 = day 1; T2 = day 2; T3 = day 3; T4 = day 4

**Fig. 2:**
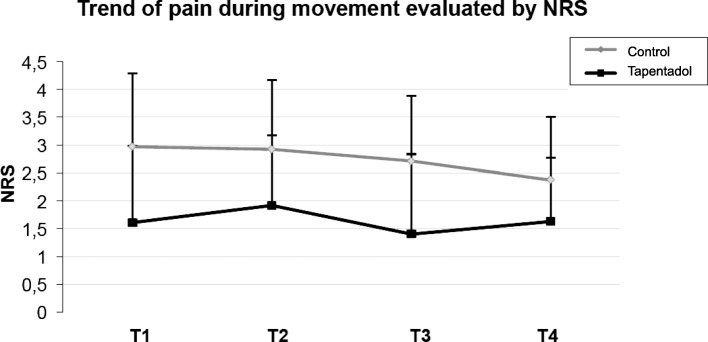
Trend of pain during movement evaluated by NRS in the two treatment groups (Grey: control; Black: tapentadol)

NRS during movement in the tapentadol group went from 1.6 on T1 to 1.9 on T2 (19% increase), followed by a decrease to 1.4 on T3, and to 1.6 on T4. Even in this case, comparison with the control group showed significantly better pain control during movement for patients treated with tapentadol at any time point evaluated (Table 1 and Fig. 2).

As for the administration of rescue analgesic doses, the rate was significantly higher in the control group compared to treatment group for all observation days, suggesting a higher efficacy of tapentadol in managing pain. Paracetamol rescue doses in the treatment group was requested by 8% of patients at T1 (2% received also morphine), 15% at T2 (3% morphine) and 9% (1% morphine) at T4, whereas in the control group it was requested by 36% of patients at T1 (7% of them received also morphine), 31% at T2 (5% morphine), and 21% at T4 (2% morphine) (Table 1).

Statistically significant differences between the two groups were also observed regarding safety and drug tolerability, i.e. post op nausea and vomit, itching, paresthesia, constipation and lipothymia (Table 2). The number of events was significantly higher in the control group compared to the treatment group. Specifically, nausea was reported in 12% of tapentadol patients vs 41% of control patients, whilst vomit was reported in 7% of tapentadol patients vs 13% of control patients (*p* < 0.05). Other collateral effects observed (Table 2) included constipation within the first 24 h by 97% of patients in the treatment group and 99% in control, a percentage that then decreased to 9 and 34% respectively on T4 (*p* < 0.001). As for itching, in the treatment group 4 cases in total (4%) were described compared to the 15 cases (14%) of the control group (*p* < 0.001).

**Table 2:** Occurrence of side effects in the two treatment groups

Side effects, n (%)	Control *n* = 105			Tapentadotol *n* = 106			*P* value
	N of patients	(%)	N of events	N of patients	(%)	N of events	
Nausea/vomiting	57	54	86	20	19	44	< 0.05
Itching	15	14	19	4	4	4	< 0.001
Paresthesia	3	3	3	1	1	1	ns
Constipation	36	34	53	10	9	12	< 0.001
Lipothymia	8	8	8	10	9	12	ns

## Discussion

The aim of the present study was to evaluate outcomes in THR patients in terms of pain management within 96 h after surgery, following the administration of tapentadol or naloxone/oxycodone in combination with ketoprofen. From the results it is clear that patients in the tapentadol group experienced a decrease in pain intensity and collateral effects compared to the control group, with statistically significant differences both in terms of NRS (at rest and during movement) and collateral effects. Consequently, these benefits helped patients adhere to the fast track protocol of rehabilitation better, which nowadays is one of the main goals in joint replacement surgery.

In particular, in the field of hip disease, the ‘experience of pain’ with its neuropathic and inflammatory components often represents a physical and mental burden to patients, to the point that it might condition their approach to surgical intervention first, and subsequently to the rehabilitation process. Tapentadol’s nociceptive and neuropathic components, that reduce the ascendant component of pain while strengthening descending inhibitory stimulation, could represent an effective option in the management of post-op pain, with a good tolerability profile (Tzschentke et al., 2014; Langford, 2016; Pergolizzi et al., 2016). Post-op management has always represented a challenge in major orthopaedic procedures such as joint replacement, and many analgesic protocols have been tested over time: in many cases opioids have been among the drugs of choice to control pain in the very first phases following surgery, but (despite providing satisfactory pain reduction) the unfavorable rate of adverse events typically associated to many opioids has been regarded as their greatest disadvantage. The search for the “ideal opioid”, i.e. a drug with sufficient analgesic power and a low rate of side effects, led to the development of new molecules: tapentadol has been recently proposed as an effective and well tolerated pain killer, suitable for use in many orthopaedic situations, including surgical-related settings (Lee et al., 2014; Lockwood & Dickenson, 2019; Chen et al., 2015).

In particular, tapentadol has reduced pharmacological interactions (low binding to plasma proteins and no impact on CPY450, no active metabolites), which makes the drug more appealing for elderly patients, who represent the majority of the THR patient population admitted to our facility, and often displaying comorbidities and/or concomitant treatments (Tzschentke et al., 2014; Biondi et al., 2015). Another relevant advantage of tapentadol is the low rate of complications associated to its use (Vadivelu et al., 2017), which is inferior to that of other similar molecules. In the present study we used oxycodone/naloxone as a comparator. Oxycodone is a traditional and “popular” opiod, whose use in the field of muscolo-skeletal diseases is well established and reported in many trials so far (Kim et al., 2018; Oppermann et al., 2016), and its combination with naloxone has been developed and marketed more recently, in the attempt of diminishing the well-known gastro-intestinal side effects reported when administering oxycodone alone (Scardino et al., 2015).

In our experience, a dosage of 100 mg twice daily (following surgery), resulted in a satisfactory management of pain for the majority of patients (250 mg being the maximum daily recommended dose).

Post-op pain, both at rest and during movement, throughout T1-T4 was always reported as being bearable, with a NRS < 2. This has certainly contributed to the quick recovery of the patients after surgery as confirmed by the fact that 50% of patients were able to stand on T0 (that is within 4 h from surgery) a value which increased to 90% on T1. Patients treated with tapentadol also displayed a better post-operative recovery, which is not only represented by lower pain scores compared to patients who received oxycodone/naloxone, but also by the absence of recurring acute pain episodes. Specifically, the lower NRS both at rest and in motion was the main factor responsible for the well being of the tapentadol patients, a factor which allowed them to perform physical rehabilitation activities early, bringing with it all the inherent advantages in terms of functional recovery.

Indeed our results are comparable to those reported by Panella et al. (Panella, 2016) who assessed pain treatment following THA in 144 patients with either tapentadol PR (50 mg twice daily) versus paracetamol (1000 mg three times daily). Patients were treated throughout rehabilitation and followed-up for three weeks, showing (in addition to a satisfactory functional recovery) a 4.3 point decrease (83%) in patients treated with tapentadol vs 2.4 points (48%) in those treated with paracetamol. Conversely another study described the administration of tapentadol starting the day before surgery, but in this case, this did not result in statistically significant advantages compared to the standard treatment with oxycodone (Haesler et al., 2017).

In our study, patients treated with tapentadol and ketoprofen on T1 reported no pain, but they did report a higher incidence of symptoms such as nausea and vomiting than on the following days thus suggesting that, at least partially, these symptoms are related to the anesthesia and post-operative analgesia. Furthermore, a higher occurrence of nausea and vomit was described in the control group at T1 and T2, which again, emphasizes the better tolerability of tapentadol compared to oxycodone/naloxone.

This observation was confirmed by the presence of a few fainting episodes, all of which occurred on T1, linked to hypotension following local anesthesia. Additionally, hemodynamic parameters of patients treated with tapentadol, such as oxygen saturation and heart rate remained stable throughout the four days of observation. Overall, the typical collateral effects of opioids such as nausea, vomiting and constipation resulted to be greatly reduced in number in the tapentadol group compared to the oxycodone/naloxone group, an observation which is supported by data present in medical literature (Coluzzi & Ruggeri, 2014; Pergolizzi et al., 2016; Sanchez de Aguila et al., 2015). The higher incidence of PONV in the oxycodone/naloxone group may also be related to the higher request for morphine as rescue-analgesia in the control group.

In brief, results from our study support the use of tapentadol in combination with ketoprofen for the management of moderate-severe pain following major orthopedic surgeries, given its effectiveness in reducing intensity of pain and its satisfactory tolerance, allowing patients to perform post-operative rehabilitation activities early, and resume ambulation quickly after surgery. Indeed, forced immobilization due to pain, post-operative fasting, nausea and vomiting all contribute to comorbidities flares and increase the onset of complications which can compromise post-operative recovery and lengthen hospital stay.

The main limitation of the present study is its retrospective design, which warrants future randomized controlled trials to confirm the findings emerged from the present analysis. Despite data being retrospectively collected, we are confident about their reliability since our Department’s dedicated personnel (i.e. anesthesiology nurses) are in charge of evaluating patient pain NRS, daily, at the same hour. Among other study limitations, the lack of a long-term follow-up is of particular note, since it does not provide any information on the treatment’s long-term outcome. It is currently thought, as suggested by data in the literature, that tapentadol offers a longer-term efficacy compared to other opioids, featuring complete tolerance at 51 days compared to 21 days with morphine (Pergolizzi et al., 2016), with no risk of addiction in the medium-long term (Buynak et al., 2015). However, there is still a substantial paucity in long-term data within the literature, with the longest studies being limited to a maximum of 2 years. Another limitation is the lack of data concerning different post-op dosages of tapentadol, since it might be argued that variations in the dose could have yielded a significantly different therapeutic response and perhaps different incidence of adverse events. Additional randomized studies are therefore needed to understand the best administration protocols to maximize the benefit/risk ratio.

## Conclusion

Based on the data of the present study, the use of 100 mg tapentadol twice a day in the management of early post-operative pain following THR surgery proved to be safe, and displayed a good tolerability profile and better results in terms of pain relief both at rest and during movement compared to another commonly used opioid such as oxycodone/naloxone. This allowed therefore an early start of the rehabilitation in the setting of a fast-track protocol following surgery.

## Data Availability

The datasets used and/or analysed during the current study are available from the corresponding author on reasonable request.
